# NETosis – Does It Really Represent Nature’s “Suicide Bomber”?

**DOI:** 10.3389/fimmu.2016.00328

**Published:** 2016-08-26

**Authors:** Shida Yousefi, Hans-Uwe Simon

**Affiliations:** ^1^Institute of Pharmacology, University of Bern, Bern, Switzerland

**Keywords:** NETosis, neutrophil extracellular traps, innate immunity, adaptive immunity, cell death

NETosis is a term that evolved following publication of an original article supposedly describing a novel form of programed neutrophil death that resulted in the formation of neutrophil extracellular traps (NETs) (1). NETosis was subsequently added to the cell death classifications, almost joining the ranks of other, better documented pathways, such as apoptosis, necroptosis, and autophagic cell death (2). Fuchs et al.’s (1) article seems to NETosis converts to be so seminal that reviewers deny publication to manuscripts in this area, which fail to reference it [Ref. (3); see Supporting Information; Peer Review Correspondence: URL: Link 1].

We have been puzzled by the ready acceptance of a proposed programed cell death in this format. Let us examine the phenomenon of NETosis as it is cited in the recent literature and see why this concept seems inconsistent with the economy of nature. NETosis was described as a death process in which the plasma membrane ruptures, allowing chromatin release following the collapse of the nuclear membrane (1). This is supposed to happen in order to rescue and protect the affected environment, and this theory has been promoted by many reputable scientific journals, including *Nature* (videos: Link 2). However, thus far, no explanation has been offered as to how the remains of neutrophils that have undergone NETosis would be eliminated under *in vivo* conditions. This lack gives pause because such residue must be expected to be potentially harmful to the host. In fact, under physiological conditions in healthy individuals, nuclear DNA release following activation of neutrophils encountering microorganisms is still controversial and the question has, in fact, been raised whether a NETosis in this format would be at all beneficial to the host (4).

We argue that under physiological conditions, NETosis would be a destructive process. NETosis implies a waste of neutrophils, but more importantly, it would mean exacerbated inflammation. We consider that it is important for neutrophils to remain viable in order to exercise their useful skills, phagocytosis of invading microorganisms and extracellular killing of pathogens by the programed release of (as we believe, mitochondrial) DNA together with granule proteins. Neutrophils can subsequently die through apoptosis (5) or, under inflammatory conditions, also by programed necrosis (6). In both cases, recognition of the dying cells by phagocytes would assure disposal without unnecessary inflammation.

Mature neutrophils are terminally differentiated white blood cells that depend on glycolysis for ATP production; hence, they can afford to lose mitochondrial DNA (mtDNA) in response to invading microorganisms. In addition, mitochondria are evolutionary endosymbionts derived from bacteria, which carry bacterial molecular motifs (7), and are considered to be master regulators of danger signaling (8). Unmethylated mtDNA, such as bacterial DNA, is the most potent activator of plasmacytoid dendritic cells (pDCs) and the type I interferon (IFN) pathway [Ref. (9–11), and our own unpublished data]. In our view, the innate immune system attempts to overcome an infection primarily with a combination of mtDNA-containing NET formation and phagocytosis. This offers the advantage that, in case of persisting infection, the mtDNA will have boosted the adaptive immune response. Furthermore, no exaggerated inflammation caused by local cell lysis occurs. In fact, the clearance of NETs occurs in an immunologically silent manner (12).

In the literature, the terms NETs and NETosis are often used indiscriminately, which is problematic. NET formation was first described by Brinkmann et al. (13). These authors observed the formation of extracellular traps consisting of DNA together with granule proteins of neutrophils, which were released upon brief stimulation with physiological agonists, such as interleukin-8 (IL-8) or lipopolysaccharide (LPS), Gram-positive and Gram-negative bacteria, and unphysiological stimuli, such as phorbol myristate acetate (PMA) that cause increases in cellular ROS levels. PMA at concentrations between 5 and 50 nM for 30 min (dose–response) and with 10 nM PMA for 10, 20, and 30 min (time course) were able to induce NET formation [Ref. (13), see Supporting Online Material]. The same was true for co-culture with bacteria, i.e., a 30-min incubation was sufficient to form NETs and kill bacteria extracellularly. The neutrophils were reported to remain viable, but the source of the released DNA was not identified at that time (13).

Owing to the apparent presence of histone reactivity in NETs, many investigators have assumed that NETs contain chromosomal DNA. This idea that NETs consist of chromosomal DNA, granule proteins, and histones has become cemented in the literature because it had already been shown that histones exhibit antibacterial activity. Thus, it just seemed to make sense. However, it is important to realize that the antibodies used to support this conclusion, i.e., that histones are present in NETs, are also known to detect DNA as well (14), especially at the high concentrations employed. In addition, the existence of extranuclear histones, namely pools of H1 and H3 in the cytoplasm, has also been reported (15).

Moreover, one has argued that the presence of citrulline-containing proteins in extracellular proteins, and presumably in NETs, is an argument in favor of NETosis. Human primary neutrophils express not only protein arginine deiminase 4 (PAD4) but also PAD2 enzymes that catalyze citrulline modification of number of proteins, most importantly fibrinogen, collagen, vimentin, and platelet actin, as well as histones (16, 17). Current evidence suggests that protein citrullination may occur extracellularly and, therefore, substrate selection by the PADs would not be limited to their subcellular localizations (e.g., not just to histones in nucleus) (16). PAD2 lacks a nuclear localization signal (18) and is highly expressed in the cytoplasm of human neutrophils (17). Interestingly, the cytoplasmic concentration of PAD2 was dramatically reduced within 30 min after stimulation with PMA and, furthermore, enzymatically active PADs were detected in supernatants of cultured, activated neutrophils (17). This observation might explain the presence of citrullinated proteins in NETs upon physiological stimulation of neutrophils (e.g., activation by platelets) *in vitro* (19) and *in vivo* (20), considering that PAD2’s main substrates are fibrin and platelets’ actin, which would be present within entangled NET structures following platelet activation in the absence of cell death. PAD2 could also citrullinate extracellular histone H3 released owing to secondary necrosis (which might occur under *in vitro* as well as *in vivo* conditions), though with lower efficiency than with PAD4 (16). It is surprising, that so far no one has investigated the potential role of PAD2 for NET formation and the protein content of NETs, respectively. Perhaps some of the discrepancies in the PAD4 knockout mouse model could be explained if we take into account the possible role of the PAD2 enzyme in extracellular protein citrullination. It was easy to pick PAD4 as the culprit owing to its nuclear localization, ignoring the fact that all PADs, including PAD4, can function extracellularly. Another reason researchers preferred PAD4 as a candidate is perhaps its specific expression in the myeloid lineage as compared to PAD2, which is ubiquitously expressed (21). This would make PAD2 less attractive as a potential commercial drug target.

Thus, considering these uncertainties, the argument that NETs contain chromosomal DNA is actually still unsubstantiated today. In fact, subsequent studies using DNA sequencing methods have established that NETs are generally composed of mtDNA [Yousefi et al. (22), McIlroy et al. (23), Wang et al. (9), and recently Lood et al. (10)]. We recognize that under certain conditions, neutrophils do release nuclear DNA. For instance, neutrophils can release nuclear DNA upon encountering bacteria capable of secreting pore-forming enzymes/toxins. This type of nuclear DNA release can occur as early as 5 min after bacterial contact (24–26). We also do not exclude the possibility that nuclear DNA originating from cells dying in the neighborhood of NETs, as a consequence of immunopathology, could subsequently bind to NETs *in vivo*. Moreover, under *in vitro* conditions, neutrophils stimulated with PMA may first form mtDNA-containing NETs before undergoing a subsequent necrotic cell death. As a consequence of necrotic cell death, nuclear DNA and histones could bind to already existing NETs. Furthermore, it should be noted that nuclear DNA released from dying neutrophils following PMA stimulation has been shown to generate a DNA cloud, rather than DNA fibers [Ref. (27); video: Link 3] (Figure 1).

**Figure 1:**
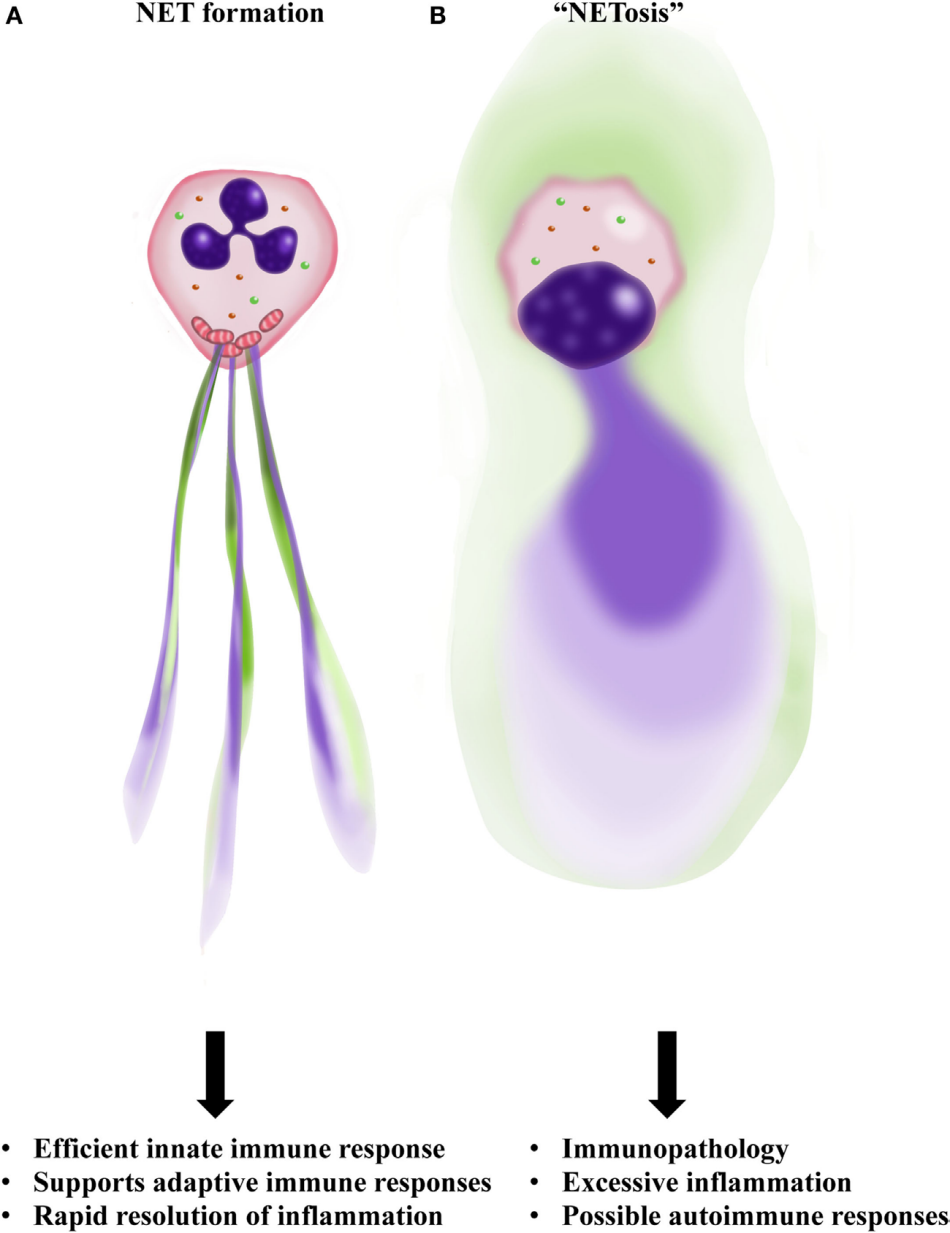
**Outline of NET formation as contrasted to “NETosis.” (A)** Neutrophils are activated by bacteria or cytokines and inflammatory mediators generated by surrounding cells. A programed release of mitochondrial DNA (mtDNA) forms a net-like structure (blue) that contains granule proteins (green), the so-called NET. **(B)** Neutrophils rupture with nuclear collapse leading to chromosomal DNA release as a DNA cloud (blue). Therefore, no real NET formation is observed as a consequence of “NETosis.” Furthermore, such a necrotic-like cell death carries the risk of releasing danger-associated molecular patterns (DAMPs), possibly resulting in excessive inflammation and autoimmunity. Illustration by Aldona von Gunten.

Since the discovery of NETs, many groups have focused on finding the molecular mechanism and origin of the DNA released. For instance, elastase-deficient mouse neutrophils were reported to be unable to form NETs (28). By contrast, NET formation has recently been reported to occur in these mice in a model of deep vein thrombosis (29). We also have not found any defect in NET formation by elastase-deficient bone marrow-derived primary mouse neutrophils activated either physiologically or with brief stimulation using low concentrations of PMA (unpublished observation). Moreover, it has been reported that NETosis could actually represent a necroptosis (30). However, on the contrary, we obtained no evidence for the involvement of the RIPK3–MLKL pathway, as would be required for genuine necroptosis (3). The conditions for forming NETs have gradually evolved from 10 to 30 min of stimulation to 3–4 h, while PMA concentrations have skyrocketed from 10–25 to 100–800 nM (31). With mouse neutrophils, NET formation after 16 h of 100 nM PMA stimulation has been reported (32). It is worth noting that even low concentrations of PMA (25 nM) are known to induce cell death due to excessive intracellular ROS levels within 2–3 h (33).

Meanwhile, our group has reported that with physiological activation or low doses of PMA (25 nM), eosinophils (34), neutrophils (22), and basophils (35) all release mtDNA combined with granule proteins within <1 h, in every case without cell death (Figure 1).

The aim of this opinion article with our inflammatory title is to raise the awareness for this problem. For more honest scientific behavior, the reviewing process is ultimately where changes will have to be made to allow opposing ideas, as long as scientifically solid, to reach the overall scientific community and to receive critical scrutiny. An established opinion is not always correct; de-construction and re-construction of theories is a part of the scientific process. As an option for defusing such long-lasting scientific controversies, it would also be appropriate to deliberately arrange that proponents of opposing viewpoints present their work at international meetings. Furthermore, there should be guidelines for the stimulation and the detection of NETs both *in vivo* and *in vitro*.

## Author Contributions

SY wrote the article. H-US corrected the article.

## Conflict of Interest Statement

The authors declare that the research was conducted in the absence of any commercial or financial relationships that could be construed as a potential conflict of interest.
